# Structure-based rationale for differential recognition of lacto- and neolacto- series glycosphingolipids by the *N*-terminal domain of human galectin-8

**DOI:** 10.1038/srep39556

**Published:** 2016-12-21

**Authors:** Mohammad H. Bohari, Xing Yu, Yehiel Zick, Helen Blanchard

**Affiliations:** 1 Institute for Glycomics, Griffith University, Gold Coast Campus, 4222, Australia; 2 Department of Molecular Cell Biology, Weizmann Institute of Science, Rehovot, Israel

## Abstract

Glycosphingolipids are ubiquitous cell surface molecules undertaking fundamental cellular processes. Lacto-*N*-tetraose (LNT) and lacto-*N*-neotetraose (LNnT) are the representative core structures for lacto- and neolacto-series glycosphingolipids. These glycolipids are the carriers to the blood group antigen and human natural killer antigens mainly found on blood cells, and are also principal components in human milk, contributing to infant health. The β-galactoside recognising galectins mediate various cellular functions of these glycosphingolipids. We report crystallographic structures of the galectin-8 *N*-terminal domain (galectin-8*N*) in complex with LNT and LNnT. We reveal the first example in which the non-reducing end of LNT binds to the primary binding site of a galectin, and provide a structure-based rationale for the significant ten-fold difference in binding affinities of galectin-8*N* toward LNT compared to LNnT, such a magnitude of difference not being observed for any other galectin. In addition, the LNnT complex showed that the unique Arg59 has ability to adopt a new orientation, and comparison of glycerol- and lactose-bound galectin-8*N* structures reveals a minimum atomic framework for ligand recognition. Overall, these results enhance our understanding of glycosphingolipids interactions with galectin-8*N*, and highlight a structure-based rationale for its significantly different affinity for components of biologically relevant glycosphingolipids.

Glycosphingolipids are ubiquitous cell surface molecules containing, at minimum, a monosaccharide joined by a glycosidic linkage to either ceramide or sphingoid^1^. These glycolipid molecules are involved in the fundamental cellular processes such as cell adhesion and signal transduction, mediated through protein-protein or protein-carbohydrate interaction^2^. Lacto-*N*-tetraose (LNT) and Lacto-*N*-neotetraose (LNnT) are the tetrasaccharides that form the core structural component of the lacto- and neo-lacto glycosphingolipid series, respectively. LNT and LNnT differ only in the type of glycosidic linkage within the non-reducing end disaccharide (Galβ1-3/4GlcNAc) (Fig. 1). Structurally, these tetrasaccharides also resemble poly-*N*-acetyllactosamine chains. The upregulation and modification of poly-*N*-acetyllactosamines into tumour-associated antigens is reported to be correlated with cancer progression^3^,^4^. The presence of galactose/*N*-acetylgalactosamine/fucose linked to the GlcNAc ring of these tetrasaccharide makes them equivalent to the core structural component of the blood group antigens. The lacto-/neolacto-series are also the carriers to some functional antigens such as blood group antigens and HNK-1 (human natural killer-1/CD57) antigens found on the hematopoietic cells and play important roles in the immune system^5^. The cell surface composition and presentation for these glycolipids vary in cancerous cells compared to normal cells, and also regulates the fate of tumour progression^6^.

Galectins are a class of lectin that recognise β-galactoside containing glycans, including the glycosphingolipids^7^. Galectin-8 is a member of the tandem-repeat galectin category, having two CRDs in tandem that are joined by an amino acid linker of variable length. Galectin-8 was first identified in prostate^8^ and lung cancer cells^9^, and later found to be widely distributed in normal tissues as well as tumour cells^10^,^11^,^12^,^13^,^14^. Similar to other galectins, galectin-8 is present in the nucleus, the cytoplasm and the extracellular space^15^. Based on the cellular context, galectin-8 regulates integrin-mediated cell adhesion, growth, and apoptosis^16^,^17^,^18^. Induction of neutrophil adhesion is a unique feature of galectin-8^16^,^18^. Galectin-8 has been shown to regulate T-cell homeostasis, exhibiting immunomodulatory and inflammatory roles with the implication in rheumatoid arthritis and uveitis^19^,^20^,^21^,^22^,^23^. Galectin-8 also plays a critical role in the capillary tube formation and endothelial cell migration *in vitro* and angiogenesis *in vivo*^24^. The ability of this lectin to regulate the bone remodelling process, by increasing expression of RANKL *in vitro* and increased bone turnover *in vivo*, can be exploited for bone-loss diseases^25^. Further, galectin-8 selectively recognises bacterial expressing blood group antigens^26^, with galectin-8*C* being shown to selectively recruit NDP52 to engulf the invading pathogens^27^.

Intracellularly, galectin-8 interactions are mainly protein-protein however it has significant interactions with glycans on the cell surface. At high concentrations, galectin-8 recognises a broad range of glycans^28^ whilst specifically recognising the blood group antigens at submicromolar concentrations^26^. The two CRDs of galectin-8, i.e. galectin-8*N* and galectin-8*C*, share ~40% sequence identity and exhibit differential glycan binding specificities. Galectin-8*N* recognises a broader spectrum of glycans compared to galectin-8*C*, and notably exhibits a preferential binding towards anionic sugars^29^. A significant contribution to the preferential binding of galectin-8 towards 3′-*O*-sulfate/3′-*O*-sialylated lactose, determined using surface plasmon resonance, was attributed to the unique binding site residues of the galectin-8*N*^29^. The majority of galectin-8 structures reported to date are for its *N*-terminal domain (galectin-8*N*): in its *apo* form, bound to lactose, and also in complex with 3′-*O*-sulfated lactose, 3′-*O*-sialylated lactose and LNFIII^30^. However, a few galectin-8*C* structures also have been reported both in the *apo* (unpublished), and bound to NDP52 peptide^31^. Structural characterisation of the full-length galectin-8 has been challenging due to high flexibility and protease susceptibility of the linker. Nevertheless, the structure of a truncated galectin-8 comprising a dipeptide linker joining the CRDs has been solved, and the truncated protein was shown to retain the neutrophil adhesion function as the intact full-length galectin-8^32^.

The tetrasaccharide LNT and LNnT are major components of human milk, providing a source of carbohydrates to the infant and also acting as physiological and immunological regulators of the intestinal tract^33^,^34^,^35^. Being rich in galactose-based glycans (lactose in particular), their interaction with galactose-recognising proteins such as galectins is of special interest. Furthermore, a recent study reports a systematic analysis of interactions of various galectin-human milk glycans, highlighting the significance of their interactions in infant health^36^. Interestingly, one of the major milk glycans found to be recognised by galectin-8 was essentially LNnT with an additional disaccharide (LacNAc) joined by a β1-3 linkage to the non-reducing end galactose. We have reported crystal structures of galectin-3 (4LBM^37^ and 4LBN^37^) and also galectin-4*C* (4YM0^38^ and 4YLZ^38^) in complex with LNT and LNnT, providing atomic details of these protein-receptor interactions. Crystal structures of galectin-9*N* bound to LacNAc dimers, that structurally resemble LNnT (differing only by the *N*-acetyl group) have also been reported (2ZHK^39^ and 2ZHL^39^). Though these galectins all bind to both these glycosphingolipid core structures, they do show evidence of fine specificity amongst them, as well as between the *N*- and *C*-terminal CRD domains in the case of tandem-repeat galectins.

In this study, we have performed crystallographic analysis to gain atomic level information pertaining to the lacto- and neolacto-series glycosphingolipids interactions with galectin-8*N*. We provide a structure-based rationale for the difference in binding affinities of LNT and LNnT, based on the observed alternative binding modes. Molecular dynamics (MD) simulations performed on the galectin-8*N*-LNT complex revealed interesting features in the binding site governing the recognition of oligosaccharide by galectin-8*N*. Comparison of the crystal structures reported in this study with previously reported galectins interacting with either these tetrasaccharides or similar ligands has led to an understanding of the nuances in binding modes and interactions across the galectin family. Crystallographic analysis of galectin-8*N*-lactose and galectin-8*N*-glycerol complexes revealed the minimum atomic framework required by galectins for ligand recognition. The atomic details of the binding mode and associated interactions of naturally occurring cell surface oligosaccharides with cell-to-cell communicating agents, such as galectin-8*N*, provide systematic understanding of the molecular phenomena.

## Results and Discussion

X-ray crystallographic structures of human galectin-8*N* were determined with bound LNT, LNnT, lactose, and glycerol at 1.58–2.00 Å resolution (Table 1).

### Galectin-8*N*-LNT complex

The galectin-8*N* CRD exhibits a typical β-sandwich comprising two anti-parallel β-sheets with the concave side housing the carbohydrate-binding site. The binding groove is formed from six beta strands labelled S1 to S6 (Fig. 2), with amino acids on strand S4-S6 forming the glycan recognition pocket (referred to as the “primary binding site”). The amino acids on strand S1-S3 form the extended binding site and are involved in recognising oligosaccharides (Fig. 2). The galectin-8*N* binding site contains a few unique residues which are either different or absent in other galectins. In galectin-8*N* the unique features are the presence of a long S3–S4 loop bearing an arginine (Arg59), the Gln47 on strand S3, Ile91 on S6, and Tyr141 on S2 (Fig. 2). These unique features potentially contribute to imparting glycan recognition specificity and may thereby affect the overall function of galectin-8. The typical binding pattern of galectin-8*N* towards disaccharides (and effectively that of typical reducing-end interactions of larger oligosaccharides) is shown by our 1.9 Å resolution lactose-bound structure (Fig. 3a, Supplementary Fig. S1), which we obtained by soaking lactose into an *apo*-galectin-8*N*-crystal (Table 1). The O4′ of lactose engages in hydrogen bonding with His65, Asn67, Arg45, and Arg69; the O6′ hydrogen bonds with Asn79, Glu89, and the glucose O3 interacts with Arg69 and Glu89. Water-mediated interactions were also observed between the galactose O3′ with the unique Arg59 and glucose O2 with Glu89 (Fig. 3b). The indirect interaction with the unique Arg59 is likely a contributing factor to the galectin-8 domain differences in binding affinity toward carbohydrates, as exemplified by galectin-8*N* having an affinity for lactose of 79 μM, compared to 440 μM by galectin-8*C*^29^. The binding mode of lactose observed in our structure is identical to previously reported galectin-8*N*-lactose bound [2YXS (unpublished), 3AP4^30^] and that of other galectin lactose complexes, as would be expected due to the evolutionarily conserved galactose recognition residues.

**Figure 1:**
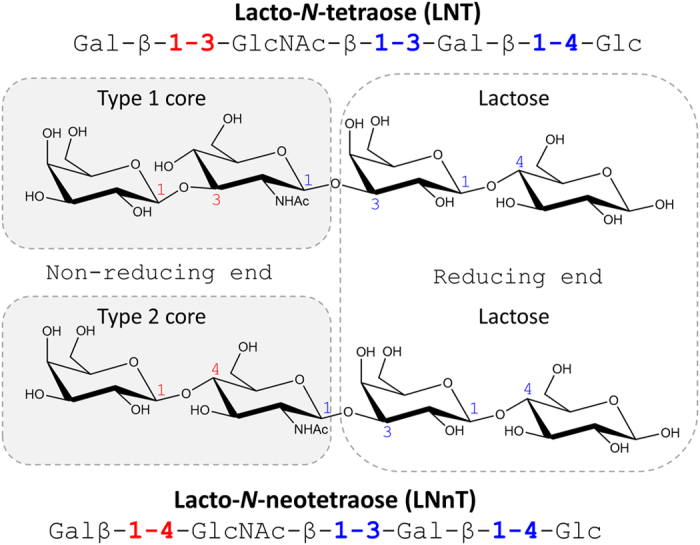
LNT and LNnT oligosaccharide structures. The LNT type 1 and the LNnT type 2 cores, as well as the reducing and non-reducing ends of the disaccharides are indicated.

**Figure 2:**
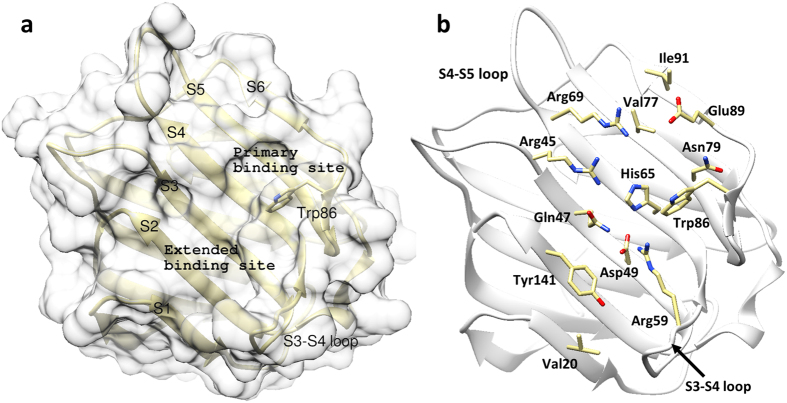
Overview of galectin-8*N* carbohydrate recognition domain. (**a**) The CRD (yellow ribbons) showing the carbohydrate binding face of the β-sandwich and the primary and extended binding regions labelled with strand S1–S6. (**b**) Depicts the amino acid residue (yellow carbon, oxygen red, nitrogen blue; stick representation) involved in glycan binding interactions.

The crystal structure of galectin-8*N*-LNT was obtained by soaking LNT into an *apo*-crystal, and determined at a resolution of 1.96 Å (Table 1). Electron density associated with the oligosaccharide was clearly visible only to the extent of revealing a disaccharide portion. Based on the structure of LNT, either the reducing end (Galβ1-4Glc) disaccharide, the non-reducing end (Galβ1-3GlcNAc; type 1 core) disaccharide or the middle disaccharide portion (GlcNAcβ1–3 Gal) (Fig. 1) would have potential to bind at the primary binding site. Of these three possible disaccharides, the non-reducing end was identified to fit the electron density map. The clear bulge of the galactose O4′ facing toward the Arg45 and Arg69, confirmed the presence of a galactose ring at the conserved galactose recognition site. Critically, the *N*-acetyl group of the GlcNAc was identified clearly in the electron density, and confirmed the location of this carbohydrate moiety (Fig. 4a, Supplementary Fig. S2). In addition, there is positive difference electron density extending at C1 of GlcNAc that implies the direction for the reducing end disaccharide (Fig. 4a). The binding of LNT to galectin-8*N* is not influenced by crystal contacts, evident by a large solvent channel alongside the ligand-binding site. Interestingly, the unit cell parameters of the galectin-8*N*-LNT complex differs from the glycerol-, lactose-, LNnT-bound structures, with the unit cell edge length *c* increased by ~10 Å whereas there is a decrease of ~2 Å in the cell edge length *a* (Table 1).

Thus in summary, the electron density map clearly shows that a galactose ring is stacked against the conserved Trp86, with the β1-3 linked GlcNAc occupying the adjacent position (that site occupied by glucose in galectin-lactose complexes) (Fig. 4a, Supplementary Fig. S2). This is the first report of the non-reducing end disaccharide occupying the primary binding site, in place of the traditionally observed reducing end disaccharide, for the tetrasaccharide LNT. This mode of type 1 core binding has not been observed previously for any other galectin. The galactose ring portion of the non-reducing end type 1 core itself makes identical interactions to that traditionally exhibited by the galactose of lactose. Significantly though, the difference in the glycosidic linkage (β1–3) of the type 1 core, as compared to β1-4 in lactose (and type 2 core), leads to variation in the placement of the GlcNAc ring and differences in overall interaction profile. Surface plasmon resonance has shown a greater binding affinity for lactose (79 μ*M*), over the type 1 core (160 μM)^29^. The GlcNAc ring is flipped by ~180° causing the *N*-acetyl group to be solvent exposed and not directly interacting with binding site residues, whilst the O4 directly hydrogen bonds with Arg69 and Glu89 and the O6 interacts with Glu89 through a water molecule (Fig. 4b). The nature of the glycosidic linkage and the placement of *N*-acetyl group are factors that influence the resulting binding affinity observed for type 1 core, which is weaker than for lactose, but stronger than for LacNAc (420 μM)^29^. Going from lactose to LacNAc results in a 5-fold weaker binding, but then changing from LacNAc to incorporate a β1,3-linkage results in an affinity that is just 2-fold weaker than lactose and ~2.6-fold stronger than for LacNAc. Conformation of the type 1 core disaccharide (Type 1 *N-*Acetyl-lactosamine) bound to galectin-1 (4XBL^40^), galectin-3 (4XBN^40^) and galectin-7 (4XBQ^40^), observed in crystal structures, also reveal an identical placement of the galactose ring, whereas the slight variation in the glycosidic torsional angle between the two rings results in different GlcNAc orientation. Given the higher average B-factor for GlcNAc (~19 Å^2^) as compared to the galactose ring (~14 Å^2^) observed in our structure, which is a similar trend to other galectin-glycan complexes, overall the binding conformation of the type 1 core appears to be comparable throughout the galectin family.

### Molecular dynamics simulations of galectin-8*N*-LNT complex

Differences in binding affinities between the two tetrasaccharides with galectin-8 reported using two independent methods, suggested a weaker affinity of LNT compared to that of LNnT, that is particularly pronounced for the galectin-8*N* domain^26^,^41^ (Table 2). For galectin-3, the affinities are relatively comparable with just a slight indication of weaker binding for LNT; but the magnitude of difference in the case of galectin-8*N* is significant (Table 2). In the case of galectin-3, both tetrasaccharides bind by their reducing ends, with the placement of the LNT non-reducing end galactose more exposed to solvent than for LNnT^37^. The implication for galectin-3 is that the different glycosidic linkages within the non-reducing end is not a dominating factor with respect to the overall binding affinities of these two tetrasaccharides. Our structure of galectin-8*N* with the LNT positioning its non-reducing end at the primary binding site is the first example of such a binding mode in galectins, and we propose that this alternative binding mode has could be the cause of the 10-fold magnitude of difference in the affinity that is reoproted between LNT and LNnT towards galectin-8*N*^29^.

Tyrosine 141 on the S2 strand is unique to galectin-8*N*, and is strikingly different to the amino acid at that position in other galectins, examples being Asn (galectin-8*C*, galectin-9*N*), Asp (galectin-1, galectin-4*N*), Gln (galectin-4*C)*, Gly (galectin-9*C*) and Ser in galectin-3. Interestingly, in galectin-8*N* this amino acid influences binding affinity of LNnT. This was demonstrated by a Tyr141Ser mutation that resulted in significant reduction in affinity for LNnT (20 μM) from that observed for wild-type galectin-8*N* (0.33 μM), as determined by fluorescence anisotropy^42^. Given the varied nature of this amino acid, that is positioned within the extended binding site region, coupled with the site-directed mutation which clearly shows it has influence on binding the tetrasaccharide then here we investigated whether the Tyr141 at this position could prove crucial in defining the binding mode and profile of galectin-8*N* towards larger oligosaccharides, and in particular whether this Tyr141 would be responsible for the alternate binding mode observed for LNT. To investigate the behaviour of LNT bound to galectin-8*N* in solution, MD simulations were carried out. For all the galectin-8*N*-LNT simulations, the starting LNT conformation used was from the galectin-3-LNT complex (4LBM^37^) where the typical behaviour of reducing end occupation of the primary binding site is exhibited (as in all other galectin-LNT structures). This approach would offer insight into why galectin-8*N* favoured instead binding to the non-reducing end of LNT. Furthermore, to investigate the influence of Tyr141 in the binding of LNT, the Tyr141Ala mutant of galectin-8*N*, and the galectin-3-LNT (4LBM) structure, were simulated. The analysis of MD results were mainly focused on the position and conformation of the non-reducing end disaccharide of LNT.

All the three systems considered here, specifically: *wt*-galectin-8*N*-LNT, Tyr141Ala-galectin-8*N*-LNT and galectin-3-LNT, showed retention of the ligand in the binding site throughout the length of the simulation. Of note is that the hydrogen bonding interactions made by the reducing end disaccharide of LNT with the conserved binding site residues showed almost 100% occupancy. Interactions made by GlcNAc with galectin-8*N* were transient due to flexibility imparted by the highly fluctuating non-reducing galactose ring (Fig. 5a). When LNT is bound via its reducing end then this most flexible galactose is positioned above the Tyr141. In the case of the *wt*-galectin-8*N*-LNT simulation the Tyr141 was one of the most flexible residues, it initially stays flat and facing the protein surface but then flips-up by about 70° after approximately 1 ns of simulation (Fig. 5a). The non-reducing end galactose then moves further away from the protein surface to accommodate the flipped Tyr141 and becomes even more flexible, and more solvent exposed. We believe that this flipping of Tyr141 induces an overall shift in the ligand positioning that results in the non-reducing end occupying the primary binding site as we reveal in the galectin-8*N*-LNnT complex structure, and in contrast to other galectins-LNT complexes.

The results of the galectin-3-LNT complex (4LBM^37^) MD simulations, that have the reducing end of LNT occupying the primary binding site, supports our hypothesis pertaining to the galectin-8*N-*LNT complex. In the case of galectin-3, there is a serine (Ser235) in place of Tyr141. As anticipated, the non-reducing end galactose in the galectin-3-LNT complex simulation was less fluctuating when compared with that observed in the *wt*-galectin-8*N*-LNT simulation (Fig. 5c). This relatively lower flexibility of the non-reducing end galactose ring possibly explains the occurrence of the reducing end galactose in the primary binding site in the galectin-3-LNT complex, unlike the alternative binding mode observed in galectin-8N-LNT complex. The presence of a smaller residue, for example the Ser in galectin-3 in place of Tyr, allows the non-reducing end galactose to relatively stabilise more during simulation and thereby weakly interact with the protein surface which eventually results in the traditional scenario of the reducing end occupying the primary binding site.

To further support the impact of the size of the amino acid at the Tyr141 location, an *in silico* Tyr141Ala mutant was also subjected to MD simulation. The small side chain of alanine should not affect the non-reducing end galactose, and therefore we should see a more stable positioning of the non-reducing end galactose. The overlayed trajectory from simulations clearly show that the galactose ring was less flexible, and occupied just one conformation for the major part of the simulation, in contrast to the situation for the *wt*-galectin-8-LNT complex (Fig. 5b). Overall, the order of flexibility of the non-reducing end galactose ring, governed by the amino acid residue positioned beneath is: galectin-8*N*-LNT > galectin-3-LNT > Tyr141Ala-galectin-8*N*-LNT. We anticipate that this order predicts that the Tyr141Ala-galectin-8*N*-LNT complex is most likely to witness the binding mode observed for LNT bound to other galectins, where the reducing end of LNT occupies the primary binding site. Thus, the MD simulation analysis supports our novel crystallographic findings and identified a critical residue Tyr141 that may potentially play a key role in determining the alternative binding mode for galectin-8*N*-LNT complex.

### Galectin-8*N*-LNnT complex

**Figure 3:**
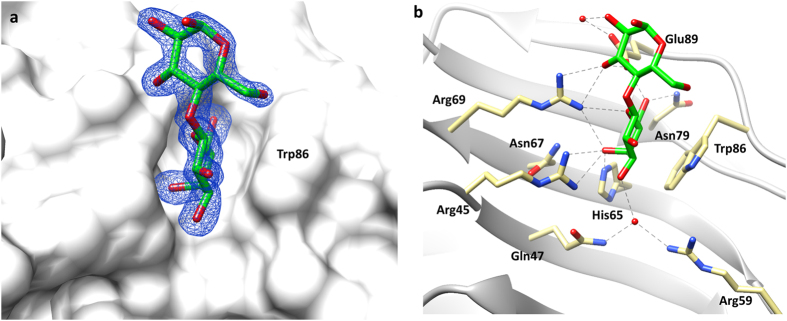
Galectin-8*N* in complex with lactose. (**a**) Electron density map (blue mesh) 2|F_o_| − |F_c_| α_c_ contoured at 1σ, for lactose (carbon green, oxygen red; stick representation) in complex with galectin-8*N* (surface representation). (**b**) Hydrogen bonding interactions (grey dashed lines) made between lactose and galectin-8*N* binding site residues (carbon yellow, oxygen red and nitrogen blue; stick representation).

**Figure 4:**
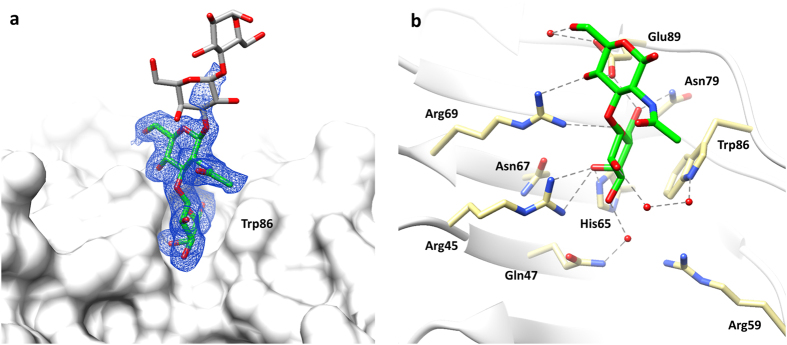
Galectin-8*N* in complex with LNT. (**a**) Electron density map (blue mesh) 2|F_o_| − |F_c_| α_c_ contoured at 1σ, for the non-reducing end disaccharide (type 1 core) portion of LNT (carbon green, oxygen red; stick representation) in complex with galectin-8*N* (surface representation). Also represented is the possible position of the reducing end disaccharide of LNT (carbon grey, oxygen red; stick representation) directed into the solvent. (**b**) Hydrogen bonding interactions (grey dashed lines) made by the non-reducing end disaccharide of LNT (green carbon, oxygen red, nitrogen blue; stick representation) with the galectin-8*N* binding site residues (yellow carbon, oxygen red, nitrogen blue; stick representation).

**Figure 5:**
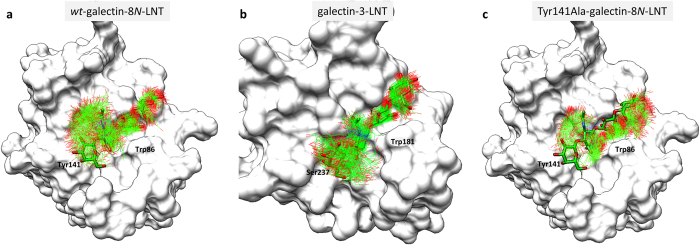
Overlay of trajectories from MD simulations. The coordinates of ligand extracted from simulations (carbon green, oxygen red, nitrogen blue; line representation) were superimposed onto the starting conformation (green carbon, oxygen red, nitrogen blue; stick representation). (**a**) Simulation of *wt*-galectin-8*N*-LNT complex. (**b**) Simulation of galectin-3-LNT complex. (**c**) Simulation of Tyr141Ala-galectin-8*N*-LNT mutant complex.

**Figure 6:**
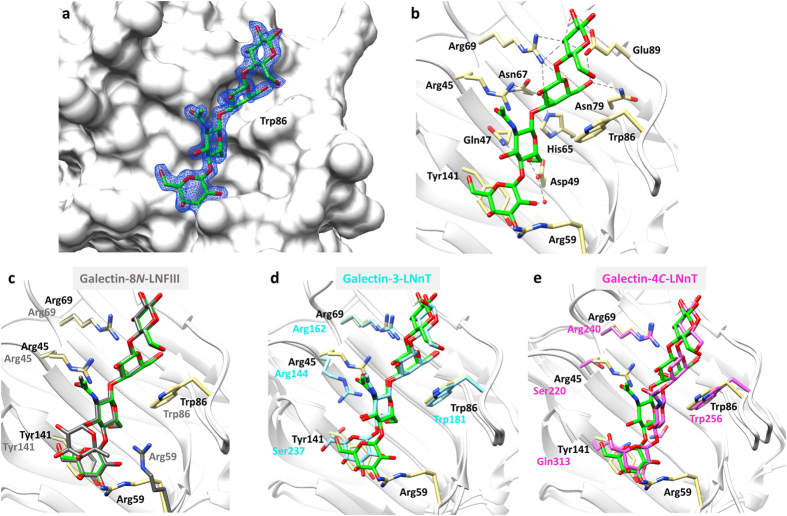
Galectin-8*N* in complex with LNnT. (**a**) Electron density map (blue mesh) 2|F_o_| − |F_c_| α_c_ contoured at 1σ, for LNnT (carbon green, oxygen red; stick representation) in complex with galectin-8*N* (surface representation). (**b**) Hydrogen bonding interactions (grey dashed lines) made by LNnT (green carbon; sticks) with the galectin-8*N* binding site residues (yellow carbon; sticks). (**c**–**e**) Superimposed (C_α_ atoms) conformation of LNnT observed in galectin-8*N*-LNnT complex with that of previously reported: (**c**) galectin-8*N*-LNFIII complex (3AP9^30^: grey carbon; sticks), (**d**) galectin-3-LNnT complex (4LBN^37^: cyan carbon; sticks) and (**e**) galectin-4*C*-LNnT complex (4YLZ^38^: magenta carbon; sticks).

The galectin-8*N*-LNnT complex was obtained by soaking LNnT into an *apo*-crystal, and the structure was determined at 2.0 Å (Table 1). The electron density maps showed unambiguous electron density for all the four sugars of the tetrasaccharide, and with the reducing end occupying the primary binding site (Fig. 6a, Supplementary Fig. S3). The lactose portion of LNnT retains the interactions observed previously in the galectin-8*N*-lactose complex (Fig. 6b). The other two sugars of LNnT extend through a β1-3 linkage into the extended binding site of galectin-8*N* contacting residues on strand S3 and S2 (Fig. 3). Due to the β1–4 linkage within the non-reducing end disaccharide (type 2 core), as opposed to the β1-3 linkage in LNT, all the four LNnT sugars stay close to the protein surface and interact with both the primary and the extended binding sites. The O6 of GlcNAc interacts with Gln47 and Asp49 (Fig. 6b) and O3 is pointing away from the binding site into the solvent. The non-reducing end galactose ring forms CH-π type interactions with Tyr141 and O2 engages in hydrogen bonding interactions with Asp49 (Fig. 6b). Arg59, is oriented away from the conserved Trp86, and may be involved in water-mediated interactions with the non-reducing galactose.

LNFIII is a branched pentasaccharide that contains LNnT as a core structure with an additional α1-3 linked fucose on the non-reducing end GlcNAc. The binding affinity of galectin-8*N* is stronger for LNFIII (3.3 μM) than for LNnT (13 μM)^29^. The crystal structure of galectin-8*N*-LNFIII complex (3AP9)^30^ revealed the structural basis for galectin-8N’s greater affinity for LNFIII than for LNnT. Essentially, the non-reducing end galactose and the branched fucose ring of LNFIII interact with the Tyr141 through CH-π type of interaction, enhancing binding affinity^29^. Overall, the conformation of the LNnT core of LNFIII in the galectin-8*N-*LNFIII complex, is identical to that observed in our galectin-8*N*-LNnT-complex (Fig. 6c). The identical ligand placement further supports that the decreased binding affinity of galectin-8*N* toward LNnT (13 μ*M*) compared to LNFIII (3.3 μ*M*) is due to the lack of a branched fucose ring on the GlcNAc in LNnT. Despite having identical ligand conformation, the orientation of unique Arg59 on the long S3-S4 loop is different in our galectin-8*N*-LNnT complex to that seen in all the reported galectin-8*N apo* and ligand-bound structures. In the galectin-8*N*-LNnT complex, Arg59 is directed towards the non-reducing end galactose, whereas in the other structures, including the galectin-8*N*-LNFIII complex, it faces towards the conserved Trp86 (Fig. 6c). In the galectin-8*N apo* and lactose-bound structures, the conformation of Arg59 appears to be uninfluenced by the presence of the ligand, and consequently stretches forward towards the conserved Trp86, further forming water-mediated interactions with the lactose. For the galectin-8*N*-LNFIII complex, the incoming ligand displaces the water molecule (located towards the 3′ position of lactose) and causes the Arg59 to move slightly away from conserved Trp86, where it then interacts with the O4 of GlcNAc. However, for the galectin-8*N*-LNnT structure, the orientation of Arg59 is unique, and this finding may hold significance in ligand specificity.

To understand the differences in binding conformation within the galectin family, comparison was performed of the LNnT conformation observed in our structure with that in galectin-3-LNnT and galectin-4*C-*LNnT complexes. In the case of galectin-3 (structure 4LBN^37^), amino acid differences (*galectin-8N given in brackets*) such as Arg186 (Ile91) on S6, Ala146 (Gln47) on S3, Ser237 (Tyr141) on S2 and importantly, the absence of the long S3-S4 loop, influence the positioning of LNnT and thereby cause a slight variation in the conformation to that found in the galectin-8*N*-LNnT complex (Fig. 6d). In contrast, the LNnT conformation in galectin-4*C*-domain (4YLZ^38^) differs to a significant extent from our galectin-8*N*-LNnT complex, possibly due to greater differences in the nature of the amino acids on strand S2. Gln313 (Tyr141 in galectin-8*N*, Ser237 in galectin-3) and Glu311 (Gly139 in galectin-8*N*, Gly235 in galectin-3) are large in size and their aliphatic chain facing the carbohydrate binding site, compared to their counterparts in galectin-3. In contrast, in the case of galectin-8*N*, Tyr141 (Gln313 in galectin-4C) although being a large amino acid, it has an aromatic side chain that stays parallel and forms CH-π type interactions with the incoming non-reducing end galactose ring. The presence of Gln313 and Glu311 in galectin-4*C* thus may cause the change in glycosidic torsion angle within the non-reducing end disaccharide, compared to that observed in LNnT bound to galectin-8*N*. These differences cause the shift in the placement of the non-reducing end galactose, leading to the overall difference in the LNnT conformation when in complex with galectin-4*C*. Thus the significance of residues present in the extended binding site governs the positioning of oligosaccharides, and anticipated to affect their overall binding strengths towards galectins.

### Galectin-8*N*-glycerol complex

**Figure 7:**
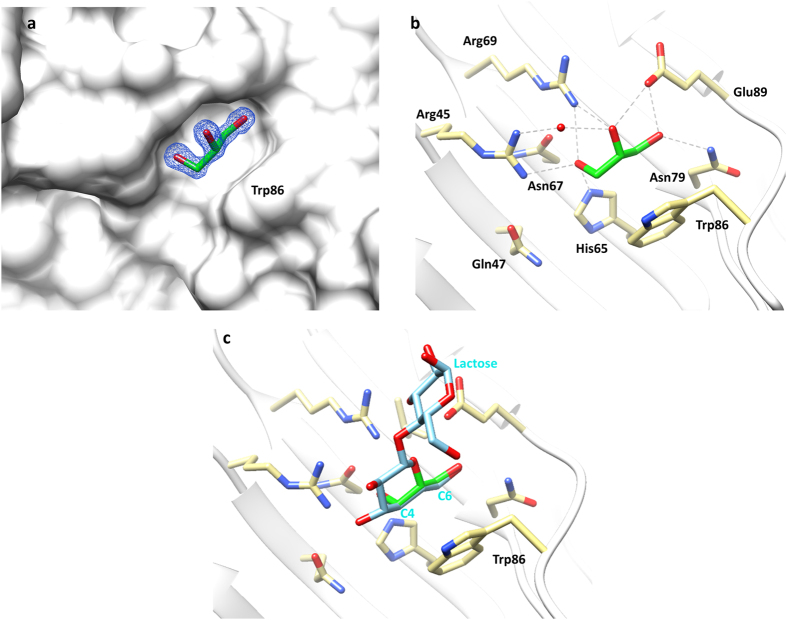
Galectin-8*N* in complex with glycerol. (**a**) Electron density map (blue mesh) 2|F_o_| − |F_c_| α_c_ contoured at 1σ, for glycerol (carbon green, oxygen red; stick representation) in complex with galectin-8*N* (surface representation). (**b**) Hydrogen bonding interactions (grey dashed lines) made by glycerol (green carbon; sticks) with the galectin-8*N* binding site residues (yellow carbon; sticks). (**c**) Superimposition of the observed glycerol conformation (green carbon; sticks) in the galectin-8*N*-glycerol complex to that of lactose conformation (cyan carbon; sticks) in the galectin-8*N*-lactose complex.

The cryoprotectant glycerol that was used during the cryo-cooling of the *apo*-galectin-8*N* crystal, soaked into the crystal and was unambiguously showed to occupy the galactose-binding site (Fig. 7a, Supplementary Fig. S4). Glycerol oxygen atoms engage in hydrogen bonding with His65, Arg69, Arg45, Asn79 and Glu89 whilst the carbon atom of glycerol makes van der Waal’s interactions with the conserved Trp86 (Fig. 7b). The glycerol hydroxyls also form water-mediated hydrogen bonds with Arg45 (Fig. 7b). The most interesting revelation from the galectin-8*N*-glycerol complex comes from alignment of the glycerol conformation observed in our structure to that of the lactose conformation and positioning within the binding site. The alignment shows an exact overlap of three carbon atoms of glycerol onto C4′, C5′ and C6′ atoms of the lactose’s galactose ring with the identical positioning of oxygen atoms (Fig. 7c). Interestingly, the position of oxygen atom of glycerol in our structure along with the presence of a water molecule (mimicking O3 of glucose in the lactose-bound structure) matches exactly with the previously highlighted hotspots for ligand recognition from the galectin-3-glycerol structure (2NMO^43^). The conformation of glycerol in our structure is also identical to that observed in other high-resolution galectin-3 structure (3ZSK^44^) and also in the galectin-4*N* structure (5DUU^45^). This indicates that the glycerol represents a moiety that exhibits key features desired for interaction by the galectins, though due to the smaller size, presence of only three hydroxyl groups and more importantly the lack of other key interactions (made by galactose) poses challenges in quantifying the glycerol binding affinity^44^. The similarity in the conformation of glycerol or water molecule location within the galactose recognition site of galectins suggests common hotspots required for ligand recognition throughout the family. This basic atomic framework together with other interactions made by galactose can be incorporated into ligand design strategies for identifying efficient binders of galectins.

### Concluding Remarks

Overall, the structures reported herein provide insight into the binding mode and interactions of lacto- and neolacto series glycosphingolipids with galectin-8*N*. The LNT and LNnT complex structure are biologically significant as they are principal components in human milk, and also form the core structural component of the blood group antigens. We demonstrate for the first time the occupancy of the primary binding site of galectin-8*N* by the non-reducing end disaccharide (thus an alternative binding mode) of the tetrasaccharide LNT, contrasting the reducing end binding traditionally observed for galectins. Hence we provide a structure-based rationale for the 10-fold weaker binding affinity of LNT towards galectin-8*N*, compared to LNnT. Amino acid differences in the extended binding site primarily governs the recognition of oligosaccharides and structures in the current study imply a preference of galectin-8*N* for neolacto-series (LNnT) over lacto-series (LNT) glycosphingolipids. MD simulations investigating the possible reasons for the alternative-binding mode for LNT to galectin-8*N*, highlighted Tyr141 as a critical residue governing the recognition of LNT. In addition, we observed a novel orientation of Arg59, which is a unique residue on the S3-S4 loop, in the galectin-8*N*-LNnT complex that may further hold significance in ligand specificity. The observed binding mode of glycerol that matches key atoms of the defining galectin ligand of galactose, highlights minimal ligand atomic features for recognition by galectins. Unique amino acid residues such as Arg59, Gln47, Ile91 and Tyr141 are other potential hotspots to not only gain affinity but also to explore specificity. Overall, taking into account critical residues in the binding site and the information about minimal atomic features for recognition, more potent and specific ligands could be designed. In all, this study not only highlights preference of galectin-8*N* towards recognising neolacto-series over lacto-series glycosphingolipids implying specific roles for the galectin-8 over other galectins but also points to structural features that can potentially be exploited for specificity in ligand design.

## Materials and Methods

### Materials

Oligosaccharides LNT and LNnT were purchased from Carbosynth Limited UK. Lactose was bought from Sigma US.

### Sub-cloning, protein expression and purification

The galectin-8*N* sequence encoded in pQE vector was amplified using a forward primer (5′-G GAA TTC CAT ATG ATG TTG TCC TTA AAC AAC C-3′) and a reverse primer (5′-CGC GGA TCC CTA CGA GCT GAA GCT AAA ACC-3′) with NdeI and BamHI restriction sites (sequence underlined) at 5′ and 3′ direction respectively. Double digestion of PCR product by NdeI and BamHI allowed sticky ends ligation into pET-3a vector resulting in pET-3a- galectin-8*N* encoding the untagged galectin-8*N*. The integrity of galectin-8*N* gene sequence inserted into pET-3a was assessed by Australian Genome Research Facility Ltd. (AGRF, Queensland, Australia). The bacterial culture grown in LB medium were induced using 1 mM IPTG (isopropyl β-D-1 thiogalactopyranoside) at room temperature for 4 h when OD of the medium reached 0.6. Cells were harvested and sonicated in a lysis buffer (10 mM sodium phosphate, 137 mM sodium chloride, 2.7 mM potassium chloride, 1.8 mM potassium phosphate; PBS) containing 1 mM PMSF (phenylmethylsulfonyl fluoride). The released protein was applied onto the lactosyl-Sepharose column and eluted using 50 mM lactose solution.

### Crystallisation, X-ray data collection and structure determination

The galectin-8*N* oligosaccharide complexes were generated by soaking *apo* galectin-8*N* crystals in the presence of the LNT/LNnT/Lactose oligosaccharides. The *apo* galectin-8*N* crystals were formed in the phosphate buffer saline at 5 mg/mL (PBS) in the microcentrifuge tube over a period of one month in the refrigerator. These crystals were used for soaking oligosaccharide dissolved in PBS at 20 mM concentration for about 18 hours. Diffraction data for all the complexes were remotely collected at the Australian Synchrotron using Blu-Ice software^46^ at 100 K with a wavelength of 0.9537 Å, and ADSC Quantum detector. The data were integrated using iMOSFLM^47^, and the point group determination and scaling of the data was performed using AIMLESS^48^. The phases were solved using Phaser^49^ with the galectin-8*N* -*apo* structure (3AP5^30^) as the search model. The model obtained was refined using REFMAC5^50^,^51^ in CCP4 program suite^52^. Visualisation and model building was done in Coot^53^. Model validation and analysis were performed by MolProbity and PDB_REDO^54^,^55^.

### Molecular dynamics simulation

GROMACS version 4.5.6^56^ was used for MD simulations with built-in AMBER99SB-ILDN force field^57^, as used for other galectins simulations^37^,^38^. Particle mesh Ewald method^58^ was employed to compute long-range electrostatics. The ligand topology and parameters were generated using acpype^59^ applied with General Amber Force Field^60^ and AM1-BCC charges^61^. Initially, the protein-ligand complex was minimized using the steepest-descent method followed by brief simulation at constant volume and then 2-ns constant pressure equilibrations. Subsequently, 100 ns production run was carried out for the complex and analysis of results was carried out using the various script provided with GROMACS.

## Additional Information

**Accession codes:** Protein Data Bank: Atomic coordinates and structure factors have been deposited with accession codes for galectin-8N CRD with bound lactose (5T7S), LNT (5T7T), LNnT (5T7I) and glycerol (5T7U).

**How to cite this article**: Bohari, M. H. *et al*. Structure-based rationale for differential recognition of lacto- and neolacto- series glycosphingolipids by the *N*-terminal domain of human galectin-8. *Sci. Rep.* **6**, 39556; doi: 10.1038/srep39556 (2016).

**Publisher's note:** Springer Nature remains neutral with regard to jurisdictional claims in published maps and institutional affiliations.

## Supplementary Material

Supplementary Information

## Figures and Tables

**Table 1:** Crystallographic data for galectin-8*N*-ligand complex structures.

	Galectin-8*N*-LNT	Galectin-8*N*-LNnT	Galectin-8*N*- Lactose	Galectin-8*N*-Glycerol
Crystal system	Orthorhombic	Orthorhombic	Orthorhombic	Orthorhombic
Space group	*P*2_1_2_1_2_1_	*P*2_1_2_1_2_1_	*P*2_1_2_1_2_1_	*P*2_1_2_1_2_1_
Unit cell	*a* = 45.40,	*a* = 47.17,	*a* = 47.61,	*a* = 47.40,
	*b* = 49.61,	*b* = 50.14,	*b* = 50.40,	*b* = 50.30,
	*c* = 80.47*	*c* = 69.86	*c* = 69.73	*c* = 69.39
Resolution (Å)	1.96	2.00	1.90	1.58
Total observations	99554 (7554)	96027 (7320)	119349 (7359)	182683 (7925)
Unique observations	12696 (929)	11695 (994)	13910 (934)	23530 (1110)
Multiplicity	7.8 (8.1)	8.2 (8.7)	8.6 (7.9)	7.8 (7.1)
Completeness (%)	93.7 (100)	99.6 (100)	100 (100)	100 (99.6)
I/σ	14.5 (11.8)	26.0 (11.4)	24.0 (8.9)	24.3 (7.3)
R_merge_ (%)	11.2 (15.3)	5.7 (14.7)	5.9 (21.9)	4.6 (19.3)
**Refinement**				
Resolution	42.23–1.96	40.73–2.00	40.85–1.90	40.73–1.58
R factor (%)	17.6	18.9	14.9	11.7
R_free_ (%)	21.8	21.7	17.9	14.7
**Number of atoms**				
Protein	1197	1178	1183	1201
Ligand	26	48	23	6
Water molecules	245	178	223	239
**Root mean square deviation**				
Bond length (Å)	0.0055	0.0086	0.0065	0.0105
Bond angle (ᵒ)	1.1742	1.3969	1.2554	1.5338
**Ramachandran plot statistics**				
Favoured (%)	98.56	97.93	98.61	97.84
Allowed (%)	1.44	2.07	1.39	2.16
**Average B-factor** (**Å**^**2**^)				
Protein	12.77	14.99	15.03	13.83
Ligand	20.95	18.47	21.81	27.32
Water	23.34	23.90	29.98	31.86
PBD ID	5T7T	5T7I	5T7S	5T7U

The values in parenthesis are for the highest-resolution shell. *Note the increase in cell edge length compared to other complexes.

**Table 2:** Binding affinities (K_d_ μM) of oligosaccharides towards galectins^29^,^41^,^61^.

Ligands	Galectin-8*N*		Galectin-3^41^
	SPR^‡^^29^	FA^‡^^42^	FA^‡^^41^
Lactose	79	3.1	2.8
LacNAc	420	9.7	1.8
LNT	140	2.1	0.97
LNnT	13	0.33	0.65

^‡^SPR - Surface Plasmon Resonance; ^‡^FA - Fluorescence Anisotropy.

## References

[b1] Chester M. A. IUPAC-IUB Joint Commission on Biochemical Nomenclature (JCBN). Nomenclature of glycolipids–recommendations 1997. European journal of biochemistry/FEBS 257, 293–298 (1998). 10.1046/j.1432-1327.1998.2570293.x 9826173

[b2] Hakomori S. Structure and function of glycosphingolipids and sphingolipids: Recollections and future trends. Biochimica et biophysica acta 1780, 325–346, doi: 10.1016/j.bbagen.2007.08.015 (2008). 17976918 PMC2312460

[b3] Demetter P. . The galectin family and digestive disease. The Journal of Pathology 215, 1–12, doi: 10.1002/path.2334 (2008). 18335458

[b4] Fukuda M., Hiraoka N. & Yeh J.-C. C-Type Lectins and Sialyl Lewis X Oligosaccharides: Versatile Roles in Cell–Cell Interaction. The Journal of Cell Biology 147, 467–470, doi: 10.1083/jcb.147.3.467 (1999). 10545492 PMC2151194

[b5] Togayachi A. . Lack of lacto/neolacto-glycolipids enhances the formation of glycolipid-enriched microdomains, facilitating B cell activation. Proceedings of the National Academy of Sciences of the United States of America 107, 11900–11905, doi: 10.1073/pnas.0914298107 (2010). 20547865 PMC2900644

[b6] Hakomori S. & Zhang Y. Glycosphingolipid antigens and cancer therapy. Chemistry & biology 4, 97–104 (1997). 9190292 10.1016/s1074-5521(97)90253-2

[b7] Perillo N. L., Marcus M. E. & Baum L. G. Galectins: versatile modulators of cell adhesion, cell proliferation, and cell death. Journal of molecular medicine (Berlin, Germany) 76, 402–412 (1998). 10.1007/s001090050232 9625297

[b8] Su Z. Z. . Surface-epitope masking and expression cloning identifies the human prostate carcinoma tumor antigen gene PCTA-1 a member of the galectin gene family. Proceedings of the National Academy of Sciences of the United States of America 93, 7252–7257 (1996). 8692978 10.1073/pnas.93.14.7252 PMC38969

[b9] Bidon N. . Two messenger RNAs and five isoforms for Po66-CBP, a galectin-8 homolog in a human lung carcinoma cell line. Gene 274, 253–262 (2001). 11675018 10.1016/s0378-1119(01)00598-4

[b10] Gopalkrishnan R. V. . Molecular characterization of prostate carcinoma tumor antigen-1, PCTA-1, a human galectin-8 related gene. Oncogene 19, 4405–4416, doi: 10.1038/sj.onc.1203767 (2000). 10980616

[b11] Bidon-Wagner N. & Le Pennec J. P. Human galectin-8 isoforms and cancer. Glycoconjugate journal 19, 557–563, doi: 10.1023/b:glyc.0000014086.38343.98 (2004). 14758080

[b12] Lahm H. . Comprehensive galectin fingerprinting in a panel of 61 human tumor cell lines by RT-PCR and its implications for diagnostic and therapeutic procedures. Journal of cancer research and clinical oncology 127, 375–386 (2001). 11414198 10.1007/s004320000207 PMC12164915

[b13] Hadari Y. R. . Galectin-8. A new rat lectin, related to galectin-4. The Journal of biological chemistry 270, 3447–3453 (1995). 7852431 10.1074/jbc.270.7.3447

[b14] Nagy N. . Galectin-8 expression decreases in cancer compared with normal and dysplastic human colon tissue and acts significantly on human colon cancer cell migration as a suppressor. Gut 50, 392–401 (2002). 11839721 10.1136/gut.50.3.392 PMC1773143

[b15] Hadari Y. R. . Galectin-8 binding to integrins inhibits cell adhesion and induces apoptosis. Journal of cell science 113(Pt 13), 2385–2397 (2000). 10852818 10.1242/jcs.113.13.2385

[b16] Nishi N. . Galectin-8 modulates neutrophil function via interaction with integrin alphaM. Glycobiology 13, 755–763, doi: 10.1093/glycob/cwg102 (2003). 12881409

[b17] Zick Y. . Role of galectin-8 as a modulator of cell adhesion and cell growth. Glycoconjugate journal 19, 517–526, doi: 10.1023/B:GLYC.0000014081.55445.af (2004). 14758075

[b18] Yamamoto H. . Induction of cell adhesion by galectin-8 and its target molecules in Jurkat T-cells. Journal of biochemistry 143, 311–324, doi: 10.1093/jb/mvm223 (2008). 18024965

[b19] Eshkar Sebban L. . The involvement of CD44 and its novel ligand galectin-8 in apoptotic regulation of autoimmune inflammation. Journal of immunology (Baltimore, Md.: 1950) 179, 1225–1235 (2007). 10.4049/jimmunol.179.2.1225 17617615

[b20] Sampson J. F. . Galectin-8 Ameliorates Murine Autoimmune Ocular Pathology and Promotes a Regulatory T Cell Response. PloS one 10, e0130772, doi: 10.1371/journal.pone.0130772 (2015). 26126176 PMC4488339

[b21] Sampson J. F., Suryawanshi A., Chen W. S., Rabinovich G. A. & Panjwani N. Galectin-8 promotes regulatory T-cell differentiation by modulating IL-2 and TGFbeta signaling. Immunology and cell biology, doi: 10.1038/icb.2015.72 (2015).

[b22] Norambuena A. . Galectin-8 induces apoptosis in Jurkat T cells by phosphatidic acid-mediated ERK1/2 activation supported by protein kinase A down-regulation. The Journal of biological chemistry 284, 12670–12679, doi: 10.1074/jbc.M808949200 (2009). 19276072 PMC2675996

[b23] Tribulatti M. V., Cattaneo V., Hellman U., Mucci J. & Campetella O. Galectin-8 provides costimulatory and proliferative signals to T lymphocytes. Journal of leukocyte biology 86, 371–380, doi: 10.1189/jlb.0908529 (2009). 19401394

[b24] Delgado V. M. . Modulation of endothelial cell migration and angiogenesis: a novel function for the “tandem-repeat” lectin galectin-8. FASEB journal: official publication of the Federation of American Societies for Experimental Biology 25, 242–254, doi: 10.1096/fj.09-144907 (2011). 20876211

[b25] Vinik Y. . The mammalian lectin galectin-8 induces RANKL expression, osteoclastogenesis, and bone mass reduction in mice. eLife 4, e05914, doi: 10.7554/eLife.05914 (2015). 25955862 PMC4424493

[b26] Stowell S. R. . Innate immune lectins kill bacteria expressing blood group antigen. Nature medicine 16, 295–301, doi: 10.1038/nm.2103 (2010). PMC2853181 20154696

[b27] Thurston T. L., Wandel M. P., von Muhlinen N., Foeglein A. & Randow F. Galectin 8 targets damaged vesicles for autophagy to defend cells against bacterial invasion. Nature 482, 414–418, doi: 10.1038/nature10744 (2012). 22246324 PMC3343631

[b28] Stowell S. R. . Dimeric Galectin-8 induces phosphatidylserine exposure in leukocytes through polylactosamine recognition by the C-terminal domain. The Journal of biological chemistry 283, 20547–20559, doi: 10.1074/jbc.M802495200 (2008). 18456665 PMC2459286

[b29] Ideo H., Seko A., Ishizuka I. & Yamashita K. The N-terminal carbohydrate recognition domain of galectin-8 recognizes specific glycosphingolipids with high affinity. Glycobiology 13, 713–723, doi: 10.1093/glycob/cwg094 (2003). 12851289

[b30] Ideo H., Matsuzaka T., Nonaka T., Seko A. & Yamashita K. Galectin-8-N-domain recognition mechanism for sialylated and sulfated glycans. The Journal of biological chemistry 286, 11346–11355, doi: 10.1074/jbc.M110.195925 (2011). 21288902 PMC3064191

[b31] Li S. . Sterical hindrance promotes selectivity of the autophagy cargo receptor NDP52 for the danger receptor galectin-8 in antibacterial autophagy. Science signaling 6, ra9, doi: 10.1126/scisignal.2003730 (2013). 23386746 PMC3713453

[b32] Nishi N. . Development of highly stable galectins: Truncation of the linker peptide confers protease-resistance on tandem-repeat type galectins. FEBS Letters 579, 2058–2064, 10.1016/j.febslet.2005.02.054 (2005). 15811318

[b33] Kunz C., Rudloff S., Baier W., Klein N. & Strobel S. Oligosaccharides in human milk: structural, functional, and metabolic aspects. Annual review of nutrition 20, 699–722, doi: 10.1146/annurev.nutr.20.1.699 (2000). 10940350

[b34] Bode L. Human milk oligosaccharides: every baby needs a sugar mama. Glycobiology 22, 1147–1162, doi: 10.1093/glycob/cws074 (2012). 22513036 PMC3406618

[b35] Asakuma S. . Physiology of consumption of human milk oligosaccharides by infant gut-associated bifidobacteria. The Journal of biological chemistry 286, 34583–34592, doi: 10.1074/jbc.M111.248138 (2011). 21832085 PMC3186357

[b36] Noll A. J. . Galectins are human milk glycan receptors. Glycobiology 26, 655–669, doi: 10.1093/glycob/cww002 (2016). 26747425 PMC4847615

[b37] Collins P. M., Bum-Erdene K., Yu X. & Blanchard H. Galectin-3 Interactions with Glycosphingolipids. Journal of Molecular Biology 426, 1439–1451, 10.1016/j.jmb.2013.12.004 (2014). 24326249

[b38] Bum-Erdene K., Leffler H., Nilsson U. J. & Blanchard H. Structural characterization of human galectin-4 C-terminal domain: elucidating the molecular basis for recognition of glycosphingolipids, sulfated saccharides and blood group antigens. The FEBS journal 282, 3348–3367, doi: 10.1111/febs.13348 (2015). 26077389

[b39] Nagae M. . Structural analysis of the recognition mechanism of poly-N-acetyllactosamine by the human galectin-9 N-terminal carbohydrate recognition domain. Glycobiology 19, 112–117, doi: 10.1093/glycob/cwn121 (2009). 18977853

[b40] Hsieh T.-J. . Structural Basis Underlying the Binding Preference of Human Galectins-1, -3 and -7 for Gal?1-3/4GlcNAc. PloS one 10, e0125946, doi: 10.1371/journal.pone.0125946 (2015). 25945972 PMC4422656

[b41] Carlsson S. . Affinity of galectin-8 and its carbohydrate recognition domains for ligands in solution and at the cell surface. Glycobiology 17, 663–676, doi: 10.1093/glycob/cwm026 (2007). 17339281

[b42] Salomonsson E. . Mutational tuning of galectin-3 specificity and biological function. The Journal of biological chemistry 285, 35079–35091, doi: 10.1074/jbc.M109.098160 (2010). 20807768 PMC2966122

[b43] Collins P. M., Hidari K. I. & Blanchard H. Slow diffusion of lactose out of galectin-3 crystals monitored by X-ray crystallography: possible implications for ligand-exchange protocols. Acta crystallographica. Section D, Biological crystallography 63, 415–419, doi: 10.1107/s090744490605270x (2007). 17327679

[b44] Saraboji K. . The Carbohydrate-Binding Site in Galectin-3 Is Preorganized To Recognize a Sugarlike Framework of Oxygens: Ultra-High-Resolution Structures and Water Dynamics. Biochemistry 51, 296–306, doi: 10.1021/bi201459p (2012). 22111949 PMC3255464

[b45] Bum-Erdene K., Leffler H., Nilsson U. J. & Blanchard H. Structural characterisation of human galectin-4 N-terminal carbohydrate recognition domain in complex with glycerol, lactose, 3′-sulfo-lactose, and 2′-fucosyllactose. Scientific Reports 6, doi: 10.1038/srep20289 (2016). PMC4734333 26828567

[b46] McPhillips T. M. . Blu-Ice and the Distributed Control System: software for data acquisition and instrument control at macromolecular crystallography beamlines. Journal of synchrotron radiation 9, 401–406 (2002). 12409628 10.1107/s0909049502015170

[b47] Battye T. G., Kontogiannis L., Johnson O., Powell H. R. & Leslie A. G. iMOSFLM: a new graphical interface for diffraction-image processing with MOSFLM. Acta crystallographica. Section D, Biological crystallography 67, 271–281, doi: 10.1107/s0907444910048675 (2011). 21460445 PMC3069742

[b48] Evans P. R. & Murshudov G. N. How good are my data and what is the resolution? Acta crystallographica. Section D, Biological crystallography 69, 1204–1214, doi: 10.1107/s0907444913000061 (2013). 23793146 PMC3689523

[b49] McCoy A. J. . Phaser crystallographic software. Journal of applied crystallography 40, 658–674, doi: 10.1107/s0021889807021206 (2007). 19461840 PMC2483472

[b50] Murshudov G. N. . REFMAC5 for the refinement of macromolecular crystal structures. Acta crystallographica. Section D, Biological crystallography 67, 355–367, doi: 10.1107/s0907444911001314 (2011). 21460454 PMC3069751

[b51] Murshudov G. N., Vagin A. A. & Dodson E. J. Refinement of macromolecular structures by the maximum-likelihood method. Acta crystallographica. Section D, Biological crystallography 53, 240–255, doi: 10.1107/s0907444996012255 (1997). 15299926

[b52] The CCP4 suite: programs for protein crystallography. Acta crystallographica. Section D, Biological crystallography 50, 760–763, doi: 10.1107/s0907444994003112 (1994). 15299374

[b53] Emsley P., Lohkamp B., Scott W. G. & Cowtan K. Features and development of Coot. Acta crystallographica. Section D, Biological crystallography 66, 486–501, doi: 10.1107/s0907444910007493 (2010). 20383002 PMC2852313

[b54] Chen V. B. . MolProbity: all-atom structure validation for macromolecular crystallography. Acta crystallographica. Section D, Biological crystallography 66, 12–21, doi: 10.1107/s0907444909042073 (2010). 20057044 PMC2803126

[b55] Joosten R. P. . PDB_REDO: constructive validation, more than just looking for errors. Acta crystallographica. Section D, Biological crystallography 68, 484–496 (2012). 22505269 10.1107/S0907444911054515 PMC3322608

[b56] Pronk S. . GROMACS 4.5: a high-throughput and highly parallel open source molecular simulation toolkit. Bioinformatics 29, 845–854, doi: 10.1093/bioinformatics/btt055 (2013). 23407358 PMC3605599

[b57] Lindorff-Larsen K. . Improved side-chain torsion potentials for the Amber ff99SB protein force field. Proteins 78, 1950–1958, doi: 10.1002/prot.22711 (2010). 20408171 PMC2970904

[b58] Essmann U. . A smooth particle mesh Ewald method. The Journal of Chemical Physics 103, 8577–8593, 10.1063/1.470117 (1995).

[b59] Sousa da Silva A. W. & Vranken W. F. ACPYPE - AnteChamber PYthon Parser interfacE. BMC Research Notes 5, 1–8, doi: 10.1186/1756-0500-5-367 (2012). 22824207 PMC3461484

[b60] Wang J., Wolf R. M., Caldwell J. W., Kollman P. A. & Case D. A. Development and testing of a General Amber Force Field. J Comput Chem 25, doi: 10.1002/jcc.20035 (2004). 15116359

[b61] Jakalian A., Jack D. B. & Bayly C. I. Fast, efficient generation of high-quality atomic charges. AM1-BCC model: II. Parameterization and validation. J Comput Chem 23, 1623–1641, doi: 10.1002/jcc.10128 (2002). 12395429

