# Supplementary material for: Structure-based rationale for differential recognition of lacto- and neolacto- series glycosphingolipids by the N-terminal domain of human galectin-8
Source: Sci Rep. 2016 Dec 21;6:39556. doi: 10.1038/srep39556 (PMC5175137; doi:10.1038/srep39556)
Supplement: Supplementary Information [file srep39556-s1.pdf]

SUPPLEMENTARY INFORMATION

Structure-based rationale for differential recognition of lacto- and neolacto- series glycosphingolipids by the *N*-terminal domain of human galectin-8

Mohammad H Bohari[1], Xing Yu[1], Yehiel Zick[2], Helen Blanchard[1*]

[1]Institute for Glycomics, Griffith University, Gold Coast Campus, 4222, Australia.

[2]Department of Molecular Cell Biology, Weizmann Institute of Science, Rehovot, Israel.

* To whom correspondence should be addressed: Associate Professor Helen Blanchard

Institute for Glycomics, Griffith University, Gold Coast Campus, 4222, Australia.

Phone: +61 7 555 27023; Fax: +61 7 555 28098 ; E -mail: h.blanchard@griffith.edu.au

SUPPLEMENTARY INFORMATION

SUPPLEMENTARY FIGURE LEGENDS

Figure S1. Structure of galectin-8N in complex with lactose. Omit electron density maps calculated from refinement with the lactose omitted from the model (2mF_o − DF_c: 1.0 σ [blue], mF_o − DF_c: 3.2 σ [green/red]) in the galectin-8*N*-Lactose complex. Red crosses indicate water molecules.

Figure S2. **Galectin-8*N* in complex with LNT.** Omit electron density maps calculated from refinement with the LNT omitted from the model (2mF_o − DF_c: 1.0 σ [blue], mF_o − DF_c: ±3.2 σ [green/red]) in the galectin-8N-LNT complex. Red crosses indicate water molecules.

Figure S3. Galectin-8*N* in complex with LNnT Omit electron density maps calculated from refinement with the LNnT omitted from the model (2mF_o − DF_c: 1.0 σ [blue], mF_o − DF_c: ±3.2 σ [green/red]) in the galectin-8*N*-LNnT complex. Red crosses indicate water molecules.

Figure S4. **Galectin-8*N* in complex with glycerol**. Omit electron density maps calculated from refinement with the glycerol omitted from the model (2mF_o − DF_c: 1.0 σ [blue], mF_o − DF_c: ±3.2 σ [green/red]) in the galectin-8*N*-glycerol complex. Chloride ion indicated by white cross, water molecules by red crosses.

SUPPLEMENTARY INFORMATION

SUPPLEMENTARY FIGURES

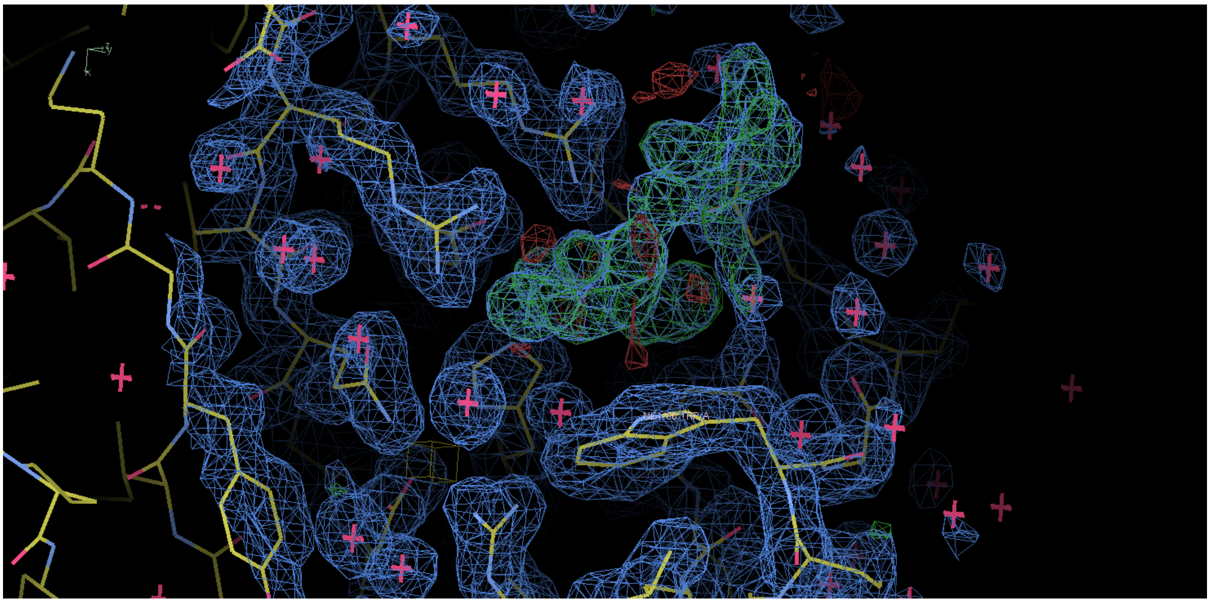

Figure S1. Omit electron density maps calculated from refinement with the lactose omitted from the model (2mF_o − DF_c: 1.0 σ [blue], mF_o − DF_c: ±3.2 σ [green/red]) in the galectin-8*N*-Lactose complex. Red crosses indicate water molecules.

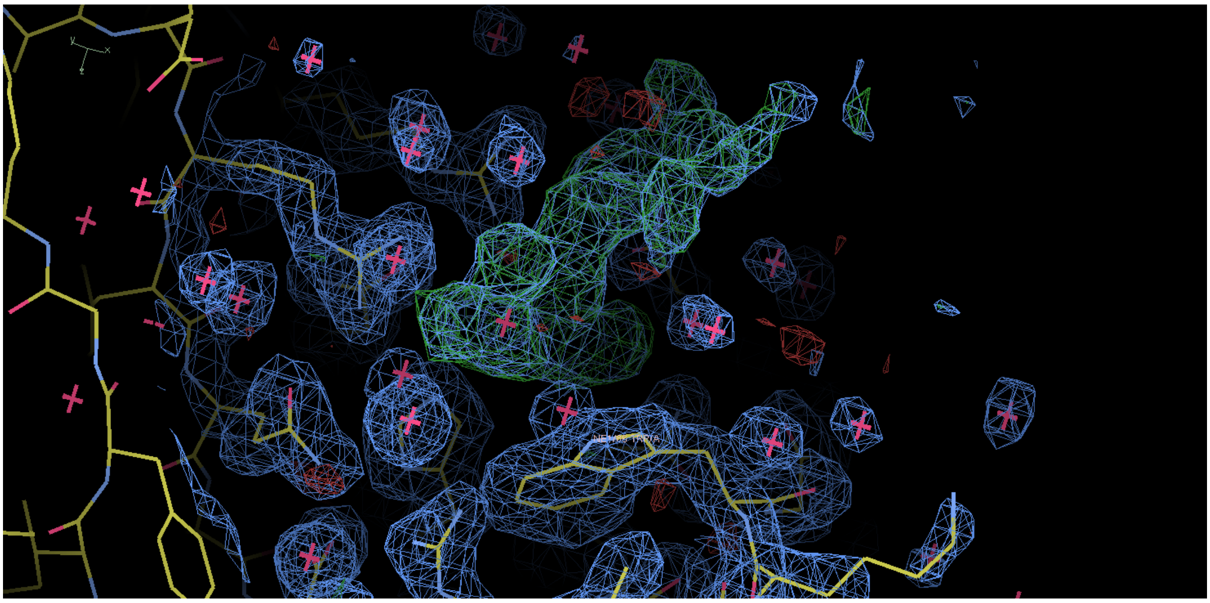

Figure S2. Omit electron density maps calculated from refinement with the LNT omitted from the model (2mF_o − DF_c: 1.0 σ [blue], mF_o − DF_c: ±3.2 σ [green/red]) in the galectin-8N-LNT complex. Red crosses indicate water molecules.

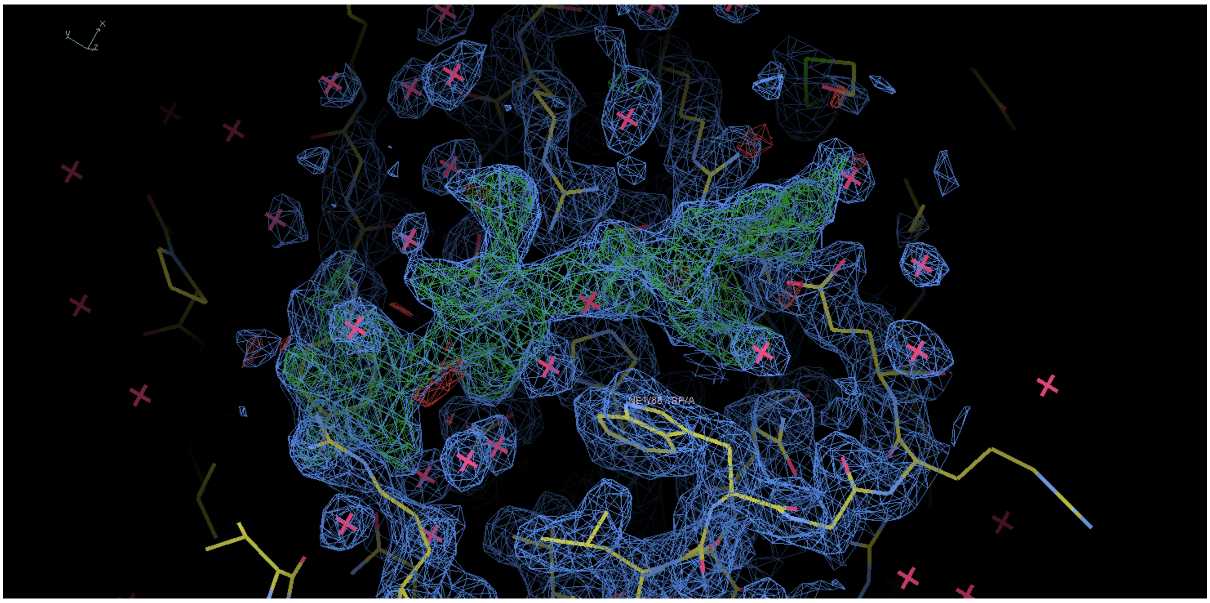

Figure S3. Omit electron density maps calculated from refinement with the LNnT omitted from the model (2mF_o − DF_c: 1.0 σ [blue], mF_o − DF_c: ±3.2 σ [green/red]) in the galectin-8*N*-LNnT complex. Red crosses indicate water molecules.

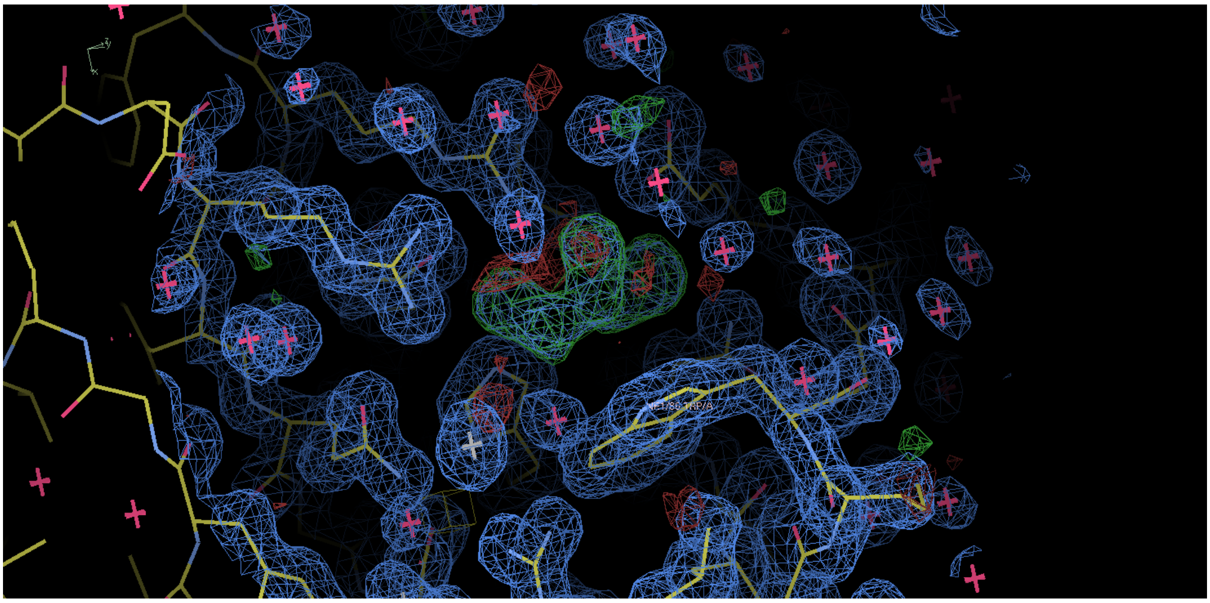

Figure S4. Omit electron density maps calculated from refinement with the glycerol omitted from the model (2mF_o − DF_c: 1.0 σ [blue], mF_o − DF_c: ±3.2 σ [green/red]) in the galectin-8*N*-glycerol complex. Chloride ion indicated by white cross, water molecules by red crosses.
